# Mitigation of renal toxicity induced by paraquat using ferulic acid: Role of inflammatory pathways

**DOI:** 10.22038/ajp.2025.26035

**Published:** 2025

**Authors:** Ali Nouri, Maryam Ghorbani, Alireza Shahriary

**Affiliations:** 1 *Clinical Biochemistry Research Center, Basic Health Sciences Institut* *e, Shahrekord Unive* *rsity of Medical Sciences, Shahrekord, Iran*; 2 *Department of Pharmacology and Toxicology, Faculty of Pharmacy* *, Baqiyatallah * *University of Medical Sciences, Tehran, Iran*; 3 *Chemical Injuries Research Center, Systems Biology and Poisonings Institute, Baqiyatallah University of Medical Sciences, Tehran, Iran*

**Keywords:** Oxidative stress, Paraquat, Ferulic acid, Renal damage

## Abstract

**Objective:**

Paraquat (PQ), a widely used herbicide, is recognized for its extreme toxicity and has been linked to numerous health concerns, particularly renal injury. The present study aimed to evaluate the effect of ferulic acid (FA) on oxidative stress and inflammation induced by PQ.

**Materials and Methods:**

Thirty-two male Wistar rats were divided into 4 groups: group 1 included healthy animals that received distilled water, group 2 received PQ (25 mg/kg, orally), group 3 received FA (100 mg/kg, orally) and group 4 received PQ plus FA. The study duration was 14 days and twenty-four hours after the last treatment, rats were anesthetized. Blood samples were taken from the heart and kidney tissue was removed. Oxidative stress markers and biochemical parameters were measured. Also, gene expression of inflammatory markers, including tumor necrosis factor-alpha (TNF-α) and nuclear factor kappa B (NF-κB), was assessed in kidney tissue using RT-PCR.

**Results:**

PQ administration increased plasma levels of biochemical parameters, decreased antioxidant enzymes activity, increased protein carbonyl and malondialdehyde (MDA) in serum and renal tissues (p˂0.05). FA administration after exposure to PQ improved all mentioned biochemical and oxidative stress markers. PQ administration was associated with increased expression of pro-inflammatory cytokines, which in turn, increased the migration of lymphocytes into the renal cells and FA treatment improved these effects.

**Conclusion:**

This study demonstrates that daily consumption of FA can serve as an effective strategy to protect the kidneys from damage caused by chemical agents such as PQ.

## Introduction

Renal toxicity is induced by a variety of medications, and other chemicals via inflammation, apoptosis and necrosis. Kidney injury from chemical agents or medications arises from direct cellular damage or immune responses triggered by the substance or its metabolites. Intracellular stress induced by toxic metabolites can cause apoptosis and necrosis and finally, cell death (Kaplowitz 2002).

Paraquat (PQ) is a highly toxic compound that causes injury to numerous tissues in the body including the liver, heart, lungs and kidneys (Liu et al. 2017; Memarzia et al.). The annual incidence of PQ poisoning has been documented to be as high as 3.8 cases per 100,000 inhabitants (Elenga et al. 2018). Mortality rates for PQ poisoning vary widely among studies. Reported mortality rates range from 33 to 91.7%, primarily due to pulmonary fibrosis and multi-organ failure (Elenga et al. 2018).

PQ likely causes renal toxicity mainly by activating oxidative stress and inflammation pathways, resulting in kidney cell damage (Wei et al. 2014). This has been extensively studied, revealing that PQ generates reactive oxygen species (ROS) via redox cycling, which leads to superoxide radicals production (Beigoli et al.). Increased ROS levels cause oxidative harm to lipids, proteins, and DNA, culminating in cell death and renal issues (Cirilo et al. 2024). PQ exposure raises pro-inflammatory cytokines like Tumor necrosis factor alpha (TNF-α) and Interleukin 6 (IL-6), which further mediate inflammation and renal dysfunction. Experimental data show elevated *TNF-α* and *IL-6* in the kidneys post-PQ treatment. PQ also activates NF-κB, a key transcription factor that boosts inflammatory gene expression, worsening renal injury through inflammatory signals (Alizadeh et al. 2022; Mohammadi Mahjoob et al. 2024).

Ferulic acid (FA), a well-known antioxidant derived from *Angelica sinensis*, *Cimicifuga heracleifolia*, and *Ligusticum chuanxiong* (Ou and Kwok 2004), has demonstrated protective effects against various toxic agents. This phenolic compound can inhibit the PI3K/Akt signaling pathway, reduce ROS, and invoke anti-inflammatory responses by modulating PPARγ and NF-κB expression (Li et al. 2021). Consequently, FA effects against neuroinflammation are associated with decreased levels of pro-inflammatory cytokines such as IL-6, TNF-α, IL-1β (Bao et al. 2019) and inducible nitric oxide synthase (iNOS) in animal models (Adeyi et al. 2023). The NF-κB pathway is typically activated by TNF-α and other inflammatory signals, leading to the expression of various genes associated with inflammation, thus promoting a state of chronic inflammation.  Administration of FA has been linked to improved renal outcomes in various experimental models, indicating its potential in therapeutic strategies for kidney injuries caused by nephrotoxins like gentamicin (Park and Han 2024). This is crucial in the context of renal protection as inflammatory processes often exacerbate kidney injury and dysfunction.

Research on the protective effects of FA against PQ-induced renal toxicity is notably limited. While several studies acknowledge the harmful effects of PQ, particularly its contribution to acute kidney injury (AKI) through mechanisms such as oxidative stress and inflammation, there is insufficient evidence specifically assessing FA efficacy in mitigating these effects. Although FA has been studied for its protective roles against various health issues, its specific effectiveness in the context of PQ-induced nephrotoxicity remains poorly explored. Prior research has primarily investigated the antioxidant and anti-inflammatory properties of FA without addressing its potential in renal toxicity induced by specific chemicals like PQ. 

Therefore, this study aimed to fill the existing gap by investigating the protective effects of FA on renal inflammation, oxidative stress, and pathological changes in rats exposed to PQ.

## Materials and Methods

**Animal groups and experimental protocols**

To evaluate the impact of PQ and FA on renal cell function, 32 male Wistar rats (6-8 weeks old) were kept under consistent conditions of 12 hr of light, 23 ± 2°C temperature, and 50% relative humidity for a week before the experiments. They were divided into four groups, with the control group receiving only normal saline and distilled water, without any drug treatment. The second group was PQ-treated rats which were orally treated with 25 mg/kg of PQ for 2 weeks (Nouri et al., 2021a). The third group was FA-treated rats which were orally treated with 100 mg/kg of FA for 2 weeks. The rats in the fourth group were also treated with PQ and then FA. All animals were anesthetized with ketamine-xylazine two weeks after treatments to gather both the kidney tissue and blood samples. A blood specimen was collected before the necropsy by puncturing the retro orbital venous sinus. The procedure of this study was accepted by the Ethics Committee of Baqiyatallah University of Medical Sciences (Ethic number, IR.BMSU.REC.1401.009).

**Assessing the serum biochemical parameters**

After collecting the whole blood, serum was provided by centrifugation at 1500 g for 10 min at 4^ο^C. The serum contents of kidney specific markers, such as creatinine (Cr), uric acid, Urea, Alanine transaminase (ALT) and Aspartate transaminase (AST) were analyzed using a biochemical kit (Pars Azmoon Co., Iran) and an auto-analyzer system (BT3000, Rome, Italy).

**Evaluating lipid peroxidation using the malondialdehyde (MDA) assay**

To determine the effects of PQ and/or FA on the lipid peroxidation, the reaction between the produced MDA and 2-Thiobarbituric acid (TBA) (Merck Co., Germany) at a wavelength of 535 nm, was measured according to the protocol proposed by Heidarian and Soofiniya. (Heidarian and Soofiniya 2011). Then, MDA levels in serum and tissue were measured using a standard curve created with MDA (Sigma) and reported as nmol/mg protein.

**Assessing protein oxidation using the protein carbonyl (PrCar) assay**

To evaluate the protein oxidation level in response to PQ and/or FA treatment, we examined the levels of carbonyl content. We used 2,4-dinitrophenylhydrazine (DNPH) to spectrophotometrically measure the contents of reactive carbonyl derivatives according to a procedure explained by Reznick and Packer (Reznick and Packer 1994). The tissue contents of PrCar in each group were displayed as nmol/mg protein.

**FRAP assay**

To assess the influence of PQ on the anti-oxidative system, we used the FRAP assay. Plasma was collected from the treated rats and the untreated control group. The plasma sample was subsequently subjected to exposure to a reagent solution that was composed of 300 mM acetate buffer, 10 mM TPTZ, which stands for trypyridyl-s-triazine, as well as 40 mM hydrochloric acid (HCl) and 20 mM ferric chloride (FeCl3). This exposure occurred over a duration of 10 min while maintaining a temperature of 37 °C. After this incubation period, the optical density, indicated as OD, was specifically measured at a wavelength of 593 nanometers. To ensure accurate results and proper calibration, FeSO4 was utilized as a standard to normalize the serum levels of FRAP.

**Evaluating the tissue antioxidant properties**

The enzymatic activity of catalase (CAT) and Superoxide dismutase (SOD) was evaluated in the kidney tissue. In this stage, 100 mg of homogenized fresh tissue of the kidney was homogenized in 9 volumes of ice-cold phosphate buffer (PBS) and then centrifuged for 10 min at 4^ο^C. The enzymatic activity of the enzymes was evaluated using the supernatant. We also used the Bradford assay to determine the protein concentrations. The activity of CAT and SOD was analyzed using H_2_O_2_ and nitro blue tetrazolium (NBT) (Sigma-Aldrich Co., St. Louis, MO), respectively.

**Evaluating the activity of glutathione peroxidase (GPx) in the renal tissue**

The fresh kidney tissues of treated rats and untreated rats were used to provide homogenates. After incubating with ethylene diamine tetraacetate, sodium azide, H_2_O_2_, and PBS, the homogenates were centrifuged at 2000 rpm and the gained supernatant was then mixed with disodium hydrogen phosphate, and (5,5′-dithiobis-2-nitrobenzoic acid) (DTNB). The activity of GPx was evaluated at the wavelength of 412 nm.

**Evaluating of reduced glutathione (GSH)**

The Ellman method was used for determining GSH levels in treated and untreated rats (Ellman 1959). If the serum contains GSH, it could reduce Ellman’s reagent to form 2-nitro-s mercaptobenzoic acid. The produced yellow color was read at 405 nm. The concentration of GSH in the serum is expressed as μmol/g tissue.

**Gene expression analysis using qRT-PCR**

Real-time PCR was performed to assess alterations in mRNA expression. Total RNA was extracted from kidney tissues of treated and untreated rats using the BIOZOL kit reagent (China) according to the manufacturer’s instructions. cDNA was synthesized from the extracted RNA using the PrimeScriptTM reagent kit (Takara Bio Inc., Japan). Subsequently, the synthesized cDNA, along with gene-specific primers, nuclease-free water, and SYBR® Green PCR Master Mix, was subjected to real-time PCR amplification using a light cycler instrument. The cycling protocol consisted of an initial activation step (30 sec at 95°C), followed by 40 cycles of denaturation (15 sec at 95°C) and combined annealing/extension (60 sec at 60°C). Gene expression was quantified relative to the reference gene *β-actin* using the 2-∆∆CT method. Primer sequences used in this study are detailed in Table 1.

**Table:** 

Reverse	Forward	Name of Primer
**5'-** **AGAGGGAAATCGTGCGTGAC-3'**	5'- AGGAAGGAAGGCTGGAAGAGA-3'	*β-actin*
**5'-CGTGAGGCTGTTTGGTTTGAG-3'**	5'-GTCTTATGGCTGAGGTCTGGTC-3'	*NF-κB*
**5'-CCGAGACTCCTCATCTGCTATT-3'**	5'-CTGGCGTGTTCATCCGTTC-3'	*TNF-α*

**Histopathologic examination**

Twenty-four hours following the final treatment of rats with PQ and FA, the animals were euthanized by xylazine and ketamine, and kidney tissue samples were fixed in 10% neutral buffered formalin. Following paraffin embedding, the resulting paraffin blocks were sectioned into 5 μm thick slices. Slides were then stained with hematoxylin and eosin (H&E) using a previously established protocol based on Bancroft and Gamble (Bancroft and Gamble 2008). Histopathological evaluation for tissue changes was performed using an Olympus BX50F4 microscope.

**Statistical analysis**

Data were analyzed using SPSS software (version 20.0, SPSS Inc., Chicago, Illinois, USA) and GraphPad Prism. Statistical significance was assessed via one-way analysis of variance (ANOVA), followed by Tukey's post hoc test for pairwise comparisons. All data are presented as mean ± standard deviation (SD) derived from three independent experiments. A P-value of less than 0.05 was considered statistically significant.

## Results

**Changes in blood biochemical parameters**

To investigate the potential of PQ to alter renal function in vivo, rats were treated with PQ (25 mg/kg) for 14 days. As depicted in Figure 1, the results demonstrated that PQ significantly increased (p<0.001) serum levels of several biochemical markers including urea, creatinine (Cr), uric acid, alanine transaminase (ALT), and aspartate transaminase (AST), relative to control animals. Following the observation of PQ's adverse effects on renal function, we sought to evaluate the protective efficacy of FA against PQ-induced toxicity. Administration of FA alone did not significantly alter serum levels of the aforementioned markers compared to controls. Notably, co-administration of PQ and FA resulted in a significant reduction (p<0.001) in serum urea, Cr, uric acid, ALT, and AST levels (Figure 1).

**Evaluating the effect of FA on PQ-induced oxidative stress in the renal tissue of the rats**

To determine whether treatment with PQ could induce oxidative stress in the kidney tissue, the amount of lipid peroxidation and protein oxidation was examined by evaluating the levels of MDA and PrCar, respectively. Our results showed that PQ (25 mg/kg) not only increased the serum and kidney levels of MDA (p≤0.05) but also elevated the amount of PrCar in the serum of rats (p≤0.001) (Figure 2), indicating that this agent could induce oxidative stress in the renal cells. One the other hand, our data showed that the amounts of renal and serum MDA and serum PrCar were significantly dropped when PQ-treated rats were exposed to FA at the dose of 100 mg/kg (Figure 2). To confirm the ability of FA in ameliorating the oxidative property of PQ, we used FRAP assay. We found that while PQ could diminish the levels of FRAP in the control group, FA at the dose of 100 mg/kg could restore this value (p≤0.01), suggesting that FA could counteract the oxidative capacity of PQ through activating the antioxidant system. 

**Figure:**
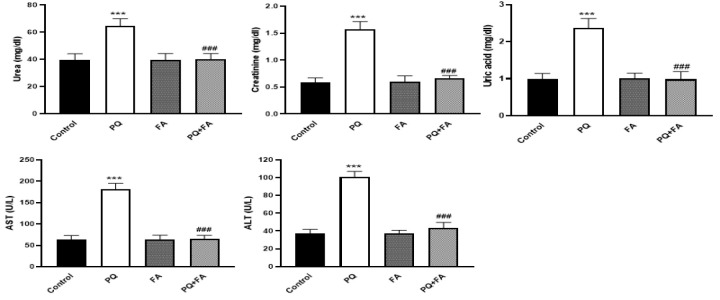


**Figure:**
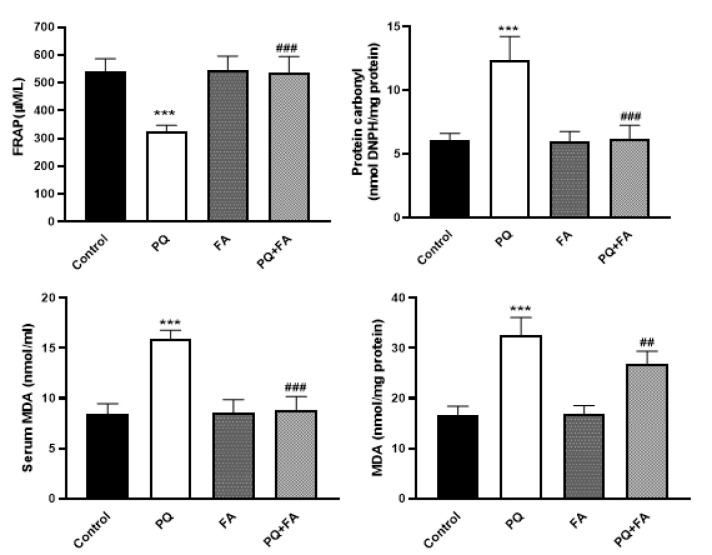


**Examining the influence of PQ and FA on the antioxidant defense system in rats**

In an organ like the kidney, the sustainment of oxidative and anti-oxidative balance is of importance. To assess the influence of PQ and FA on the antioxidant capacity of the rats, we evaluated the enzymatic activity of CAT, GPx, and SOD as well as the tissue levels of GSH. In agreement with the impact of PQ on the oxidative-related factors, the statistical analysis showed that PQ significantly abrogated the activity of antioxidant-related enzymes in the renal tissue of the animals, as compared to the control rats (Figure 3) (p≤0.001). On the other hand, our results revealed that FA could remarkably protect the renal tissue from PQ, probably through reinforcing the activity of SOD, GPx, and CAT (Figure 3). In comparison with PQ-treated rats, when rats were treated with PQ-plus-FA, we found that not only the enzymatic activity of CAT, SOD, and GPx increased, but also there was an elevation in the tissue levels of GSH (Figure 3) (p≤0.01).

**The effect of PQ and FA on the inflammatory biomarkers in the renal tissue of rats**

We evaluated the gene expression of pro-inflammatory factors using qRT-PCR analysis. Results of this work showed that PQ significantly increased the expression of *TNF-α* and *NF-κB* in the kidney of animals, as compared to the control rats (Figure 4) (p≤0.001). Additionally, when we treated the PQ-treated rats with FA, we found that there was a significant reduction in the gene expression levels of the aforementioned genes (Figure 4) (p≤0.001).

**Figure:**
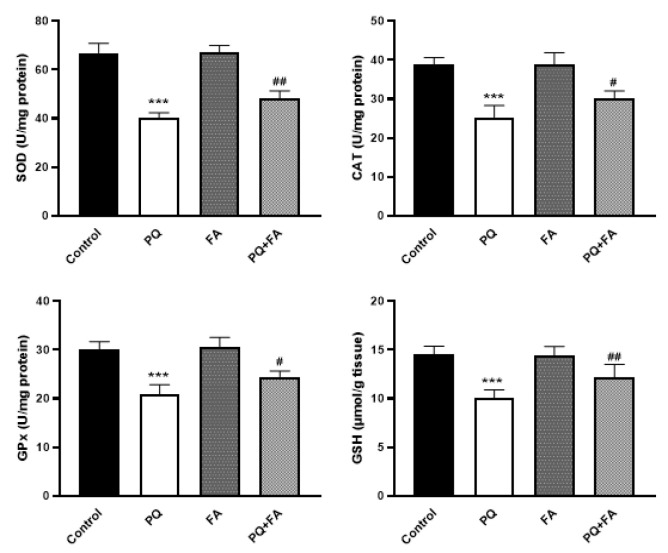


**Figure:**
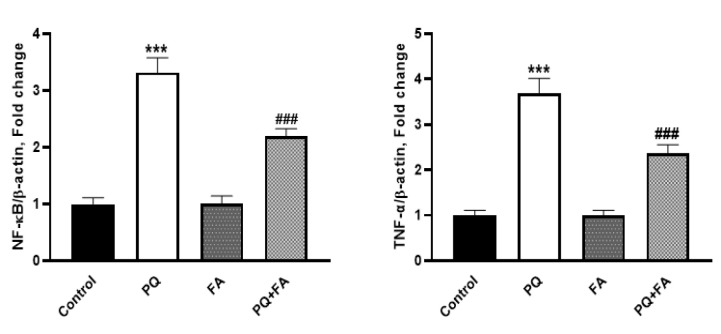


**The effect of PQ and FA on the histopathological changes of the renal tissue of the rats**

When it became evident that PQ could induce pro-inflammatory responses in the renal tissue, we did histopathological examinations. The results showed specific changes in the kidney of animals treated with PQ. As compared to the control group (Figure 5A), the signs of lymphocyte infiltration were evident in the tissue of PQ-treated animals (Figure 5B), which could be probably due to the PQ-mediated induction of inflammatory responses. To confirm that the infiltration of lymphocytes was due to PQ, rats were treated with FA-only and the results of the histopathological examination showed normal renal cells without any signs of lymphocyte infiltration (Figure 5C). Moreover, treatment with FA after exposure to PQ remarkably reduced the number of infiltrated lymphocytes in the renal tissue (Figure 5D).

**Figure:**
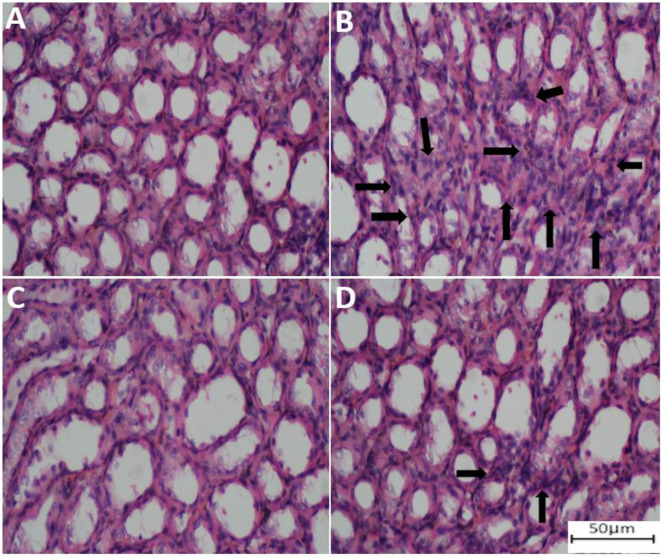


## Discussion

PQ is widely used around the world as a potent herbicide (Liu et al. 2017). ROS and nitrite radicals, are produced through the paraquat PQ metabolism pathway , can cause sever oxidative damage to macromolecules such as proteins and lipids (Chen et al. 2010). Besides lung toxicity, 

the major target organ, it has been revealed PQ causes kidney damage with high concentrations in the proximal tubules of the kidney. Various studies have shown that PQ-induced nephrotoxicity is associated with oxidative stress, inflammation, apoptosis, and direct damage to the renal tubules (Kimbrough 1974). 

The results of this study indicated that the administration of 25 mg/kg PQ to animals for 14 days resulted in deleterious effects on hepatic and renal function. This was evidenced by significant elevations in serum creatinine (Cr), uric acid, urea, aspartate aminotransferase (AST), and alanine aminotransferase (ALT) levels. The association between elevated serum levels of these biomarkers and kidney injury is well-established in previous literature (Giridharan et al. 2017). Prior research has demonstrated the potential of FA to mitigate the toxicity induced by various drugs and toxicants.(Erseckin et al. 2022; Nouri et al. 2022). Remarkably, primary results of this study also displayed that FA was efficacious in improving kidney function in animals after feeding of PQ with a substantial decrease in the serum contents of kidney and liver associated biomarkers. One of the primary mechanisms by which FA exerts these protective effects is through its ability to scavenge free radicals. By neutralizing ROS, FA helps mitigate oxidative damage to cellular components such as lipids, proteins, and DNA, thereby preserving cellular integrity and functionality.

Oxidative stress induction is considered a critical mechanism by which PQ may elicit tissue damage (Ghasemi et al. 2023; Nouri et al. 2021a; Nouri et al. 2021b). The overproduction of free radicals is known to activate autophagy and apoptotic pathways in target cells, leading to tissue injury (Amin et al. 2025; Jung et al. 2020). Results of present study showed that PQ induced lipid peroxidation as well as protein oxidation, as discovered by the increase in the serum and tissue contents of MDA and serum protein carbonyl level. Given the toxic effects of PQ on the function of the renal cells, we aimed to assess whether FA, a phenolic compound that is found widespread in plant cell walls, could protect the renal cells from PQ-induced oxidative stress. Our results showed that when PQ-treated rats were exposed to FA, this agent successfully reversed PQ-induced damages. We found that in the serum of PQ-plus-FA-treated rats, there was a significant reduction in the oxidative markers as well as MDA and protein carbonyl. More interestingly, while PQ abrogate the antioxidant system in the renal cells of the animals, FA elevated the serum levels of both FRAP and GSH, two indicators of the antioxidant defense system. When it comes to oxidative system, SOD, CAT, and GPx are the most well-known enzymes that their activity reduce the amount of free radicals in the renal cells (Muradian et al. 2002). Through degrading the free radicals, these enzymes sustain the integrity of renal cell membrane and prevent mitochondrial-mediated cell apoptosis (Muradian et al. 2002). Regard to oxidative injury of PQ, the catalytic activity of these enzymes also significantly dropped in rats, suggesting that PQ induced prolonged oxidative stress in the renal cells by preventing the activation of the antioxidant system. Contrarily, poisoned rats treated with FA showed to have activated forms of SOD, CAT, and GPx. Mahmoud et al. have also indicated that FA could counteract the toxicity of methotrexate in the animals through elevating the activity of GSH, SOD, GPX, and CAT (Mahmoud et al. 2020). In aflatoxin B1-induced liver injury in rats, FA also showed to could increase the activity of anti-oxidant-related enzymes and improve the histopathological feature of the liver (Wang et al. 2021). Subsequently, it is reasonable to assume that perhaps FA prevents the toxic effects of PQ on the renal cells through reinforcing the antioxidant system. Additionally, FA has been shown to enhance the activity of endogenous antioxidant enzymes, such as SOD, CAT, and GPx. By upregulating these enzymes, FA contributes to an increased antioxidant defense system, promoting cellular resilience against oxidative challenges. This modulation of the endogenous antioxidant system is particularly significant in conditions associated with inflammation, where oxidative stress is a common feature.

Activation of oxidative stress in the renal cells, besides to its deleterious effects on the function of the cells, could activate pro-inflammatory responses (Amin et al. 2024; Escobar et al. 2009). Once cellular oxidative stress is propagated, the transcriptional activity of NF-κB is activated, facilitates the expression of numerous of genes, in particular inflammatory cytokines through translocating into the nucleus (Timucin and Basaga 2017). In the present study, we found that PQ could also increase the expression of NF-κB in the kidney tissue of the animals and in turn increased the expression of TNF-α through integrating with apoptotic-related proteins, such as FAS, TNF-α could induce tissue necrosis (Berrrrpohl et al. 2007). Moreover, TNF-α increase the migration of lymphocytes into the injured site to propagate inflammatory responses (Mbow et al. 1994). However, FA downregulated the expression of pro-inflammatory cytokines in renal cells. Considering our findings, it has also been shown that FA could also ameliorate the toxic effects of immunosuppressant drugs and improve the morphology of the renal cells through reducing tissue apoptosis (Nouri et al. 2022). Moreover, FA exhibits anti-inflammatory properties by inhibiting the activation of NF-κB, a transcription factor that plays a crucial role in the inflammatory response. By dampening the activation of NF-κB, FA can reduce the expression of pro-inflammatory cytokines, thereby contributing to an overall decrease in inflammation-related tissue damage.

Due to the 60-70% mortality rate from PQ poisoning and the lack of an effective antidote, investigating natural compounds such as FA offers a promising path for therapy development. FA potential to mitigate oxidative stress and inflammation linked to PQ may improve outcomes for affected individuals.

In the current study, the effects of PQ and FA on other oxidative biomarkers and biochemical parameters were not evaluated. Specifically, immunohistochemical analysis of proteins and genes related to renal function, including KIM1, IL18, caspase-3, glutathione reductase, and glutathione-S-transferase, was not performed. Given the role of these factors in oxidative stress, their assessment is recommended in future investigations.

FA antioxidant and anti-inflammatory properties, along with its ability to enhance kidney function and reduce inflammation stemming from PQ exposure, position it as a promising candidate for therapeutic strategies aimed at mitigating PQ toxicity. 

Further research and clinical trials would be necessary to establish its efficacy and safety in humans suffering from PQ poisoning.
